# Physical Examination for the Detection of Pulmonary Hypertension: A Systematic Review

**DOI:** 10.7759/cureus.18020

**Published:** 2021-09-16

**Authors:** Richard A Shellenberger, Komal Imtiaz, Niranjana Chellappa, Lakshmi Gundapanneni, Caleb Scheidel, Rishin Handa, Aparna Bhat

**Affiliations:** 1 Internal Medicine, Saint Joseph Mercy Ann Arbor Hospital, Ann Arbor, USA; 2 Cardiovascular Medicine, Memorial Healthcare System, Hollywood, USA; 3 Pulmonary and Critical Care, Ascension Macomb-Oakland Hospital, Warren, USA; 4 Statistics, Saint Joseph Mercy Ann Arbor Hospital, Ann Arbor, USA; 5 Cardiology, Tower Health Medical Group, West Reading, USA; 6 Pulmonary and Critical Care, Cleveland Clinic, Cleveland, USA

**Keywords:** pulmonary hypertension, physical examination, clinical diagnosis, systematic review and meta analysis, evidence base medicine

## Abstract

We performed a systematic review to determine whether the physical examination can reliably assist in the diagnostic approach for patients suspected of having pulmonary hypertension (PH). Using dual extraction, two investigators independently searched PubMed, Ovid MEDLINE, Cochrane Library, and Embase for studies that compared physical examination findings with a right heart catheterization, from inception until July 10, 2021. We obtained data from four studies that evaluated physical examination findings in patients receiving a right heart catheterization to diagnose PH. Pooled diagnostic odds ratios (DOR) were calculated for right ventricular heave, a loud pulmonic component of the second heart sound (P2), jugular venous pressure (JVP) 3 cm above sternal angle, and a palpable P2.

Three physical examination findings had DOR that supports the diagnosis of PH: the JVP > 3 cm above the sternal angle (5.90, 95% CI 2.57, 13.57), a loud P2 (2.91, 95% CI 1.38, 6.10), and a right ventricular heave (2.78, 95% CI 1.12, 6.89). The palpable P2 had a DOR less than one and was not able to be conclusive in diagnosing PH.

Our systematic review found a small body of evidence supporting the use of physical examination tests in the diagnostic evaluation of pulmonary hypertension. The JVP > 3 cm above the sternal angle was the most accurate physical examination sign for the diagnosis of PH. Larger cohort studies using a combination of tests may shed more light on the role of the physical examination in the diagnosis and early detection of pulmonary hypertension.

## Introduction and background

Pulmonary hypertension (PH) is a pathophysiologic condition that is most often secondary to pulmonary, cardiac, or other systemic diseases. The World Symposium on Pulmonary Hypertension Task Force and the World Health Organization classify PH into five groups based on similar pathophysiologic and hemodynamic characteristics: (1) pulmonary arterial hypertension (PAH); (2) PH due to left heart disease; (3) PH due to lung disease and/or hypoxia; (4) PH due to pulmonary artery obstruction; and (5) PH with unclear or multifactorial mechanisms [1,2]. These groups are further subdivided into 27 subgroups. Given the diversity of disorders causing PH, it is not surprising to have inconsistent reporting on the global incidence of PH. Prevalence data has been reported as 97 cases per 1,000,000 in the United Kingdom [3].

The clinical importance of PH is apparent given the natural history of this condition. Early symptomatology is often benign and non-specific, yet disease progression can lead to significant functional limitations, decompensated right heart failure, and poor survival [4]. New York Heart Association functional class and five-year survival in a cohort of 2039 patients with newly or previously diagnosed World Health Organization group 1 PH were studied in the registry to evaluate early and long-term management of pulmonary arterial hypertension; group 1 PH is also known as pulmonary artery hypertension [4]. In both newly and previously diagnosed groups of patients, the five-year survival continuously worsened as New York Heart Association (NYHA) functional class increased from I to IV (43.8% in the newly diagnosed and 27.2% in those previously diagnosed class IV NYHA) [4]. The significance of these outcomes is concerning given the improvement in survival with current advances in treatment when PH is detected at earlier stages. Diagnostic strategies to diagnose PH in earlier stages may lead to improved disease-specific mortality. Across the spectrum of all NYHA classes, the newly diagnosed group of patients in this registry were more likely to begin therapy as compared to those in the previously diagnosed group. This finding validates the potential benefit of an early diagnosis. Unfortunately, most patients with PH are diagnosed at later stages. A national registry from France confirmed that 75% of patients with group 1 PH have New York Heart Association class III or IV at the time of diagnosis [5]. Patients with early-stage PH often have minimal symptomatology; however, it is not clear whether these patients have physical findings which may lead to earlier detection. A strategy to reliably identify patients at high risk to develop PH, with a clinical examination, may have a favorable impact on long-term survival for this disease. 

The physical examination has been long been advocated in clinical practice guidelines as part of the initial evaluation for the detection and diagnosis of patients suspected to have PH [3,6-8]. To our knowledge, there is little consensus on an evidence-based approach for the use of physical examination in the diagnosis of pulmonary hypertension. We designed a systematic review to answer our question of whether physical examination can reliably detect the presence of pulmonary hypertension or help to exclude the diagnosis.

## Review

Methods

Data Sources and Searches

Our systematic review is reported in accordance with the Preferred Reporting Items for Systematic Reviews and Meta-Analyses statement [9]. We began by writing a patient, intervention, comparison, outcomes, and study design (PICOS) question. Medical subject heading terms and text words based on common indexing practices were employed for our medical literature search. Search terms were compiled and tested repeatedly to produce sensitive searches and capture potentially relevant publications. The databases searched from inception to July 10, 2021, were PubMed, Ovid MEDLINE, Cochrane Library, and Embase, without language restrictions. Two investigators (RAS and AB) independently performed searches to include the following terms: pulmonary hypertension, pulmonary arterial hypertension, clinical diagnosis, diagnosis, physical examination, and bedside examination. Author and reference tracking was used to identify additional studies. 

Study Selection

**Figure 1:**
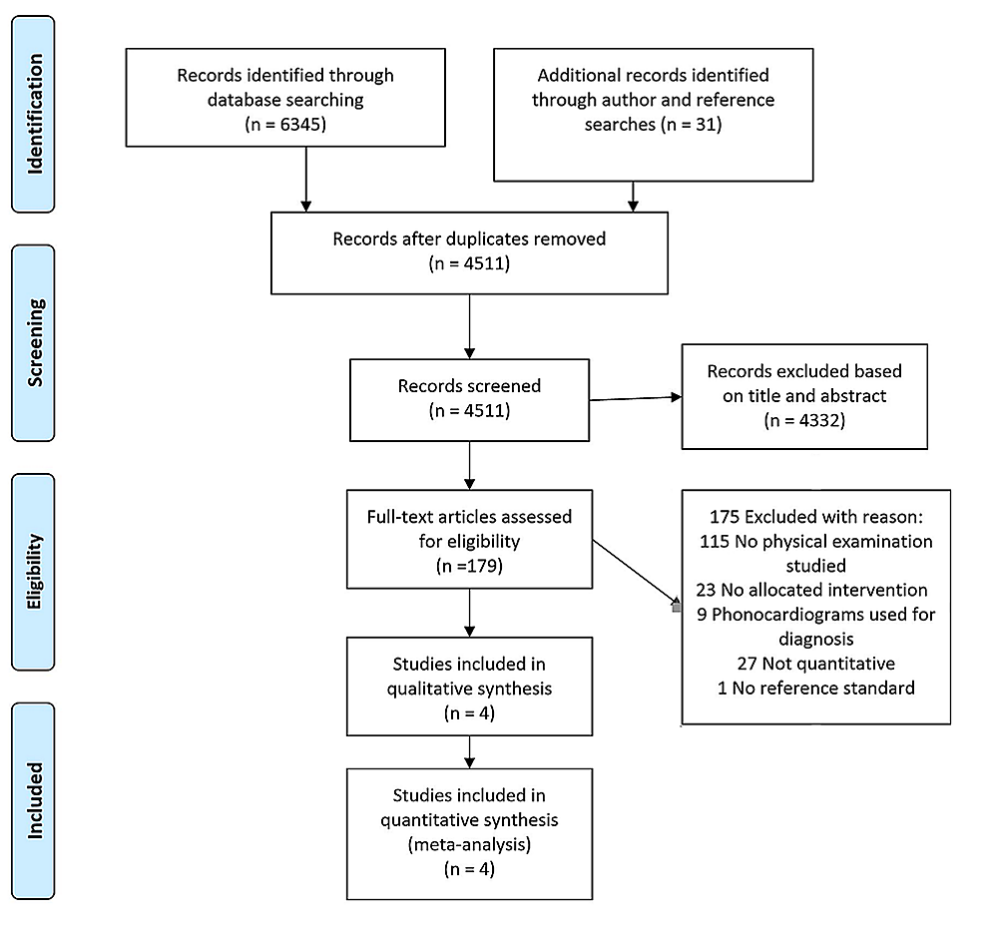
PRISMA Flow Diagram PRISMA: Preferred Reporting Items for Systematic Reviews and Meta-Analyses

With the use of reference management software, we collected initial references in citation files, removed duplicates, and two investigators (RAS and AB) began the screening from titles and abstracts for eligibility criteria (Figure 1). These studies were then screened by full-text review by the same two investigators for potential inclusion. Included studies were required to be comprised of patients having a physical examination performed prior to receiving a right heart catheterization as the "gold standard" for the diagnosis of PH. The right heart catheterization is a class 1, grade C evidence according to the European Society of Cardiology/European Respiratory Society for the diagnosis of PH [7]. We used the standard definition of a mean pulmonary artery pressure greater than or equal to 25 mmHg for establishing the diagnosis of PH [7]. Disagreements among investigators throughout the screening process were reconciled through discussion and consensus. Included trials had only human subjects who were at least 18 years old, and not limited to a specific time frame. The studies with only an abstract were excluded. Studies that did not compare physical examination findings to a right heart catheterization finding were excluded. The full study protocol is available in the Appendices.

Data Extraction, Risk of Bias, and Quality Assessment

Two investigators (RAS and AB) independently reviewed each included study, and all the data was extracted, including tables and figures (Figure 1). Disagreements were addressed by consensus and by a third reviewer (RH). Data extracted from studies included primary author, time period of the study, year of publication, patient baseline characteristics, patient demographics, research setting, research methods, and diagnostic findings. Interobserver agreement for our study selection had a kappa statistic of 0.43. Assessment of the quality and risk of bias of all included studies was independently performed by RAS and RH, employing the quality assessment tool for the observational, cohort, and cross-sectional studies available from the National Institute of Health [10] and the Cochrane collaborative tool for assessing the risk of bias, respectively [11].

Data Synthesis and Analysis

We were unable to perform a meta-analysis due to the small number of studies and included patients meeting our inclusion criteria. Our systematic review identified four physical examination findings for comparison: right ventricular (RV) heave; loud pulmonic component of the second heart sound (P2); jugular venous pressure (JVP) elevation > 3 cm above the sternal angle; and a palpable P2. Sensitivity, specificity, and likelihood ratios were calculated for each individual study as well as pooled data and its related examination findings (Table 1). Reporting pooled sensitivity and specificity can enhance the accuracy of data with small sample sizes [12]. Pooled diagnostic odds ratios (DOR) were estimated using a univariate random-effects model following the approach of DerSimonian and Laird [13]. We are reporting the accuracy of our diagnostic tests as DOR as this metric is a reliable estimate of the discriminative power of diagnostic tests and for a comparison of the diagnostic accuracy of between two or more tests without dependence on the disease prevalence (Table 2) [14]. 

**Table 1:** Pooled Diagnostic Odds Ratios and Likelihood Ratios RV: right ventricular; JVP: jugular venous pressure; P2: pulmonic component of the second heart sound

Physical examination	Pooled diagnostic odds ratio (95% CI)	Pooled positive likelihood ratio (95% CI)	Pooled negative likelihood ratio (95% CI)
RV Heave	2.78 (1.12, 6.89)	2.12 (1.01, 4.47)	0.76 (0.64, 0.90)
Loud P2	2.91 (1.38, 6.10)	1.50 (0.96, 2.33)	0.65 (0.49, 0.86)
Palpable P2	0.81 (0.37, 1.77)	0.87 (0.54, 1.43)	1.08 (0.80, 1.43)
JVP 3 CM	5.90 (2.57, 13.57)	2.47 (1.41, 4.33)	0.42 (0.31, 0.57)

**Table 2:** Quality Assessment Y: yes; N: no; CD: cannot determine; NA: not applicable

Criteria	Pilatis et al., 2000 [15]	Solverson et al., 2016 [16]	Colman et al., 2014 [17]	Braganza et al., 2019 [18]
1. Was the study question or objective clearly stated?	Y	Y	Y	Y
2. Was the study population clearly and fully described, including a case definition?	Y	Y	Y	Y
3. Were the cases consecutive?	Y	Y	Y	Y
4. Were the subjects comparable?	Y	CD	CD	Y
5. Was the intervention clearly described?	Y	Y	Y	Y
6. Were the outcome measures clearly defined, valid, reliable, and implemented consistently across all study participants?	Y	CD	Y	Y
7. Was the length of follow-up adequate?	NA	NA	NA	NA
8. Were the statistical methods well-described?	Y	CD	Y	Y
9. Were the results well-described?	Y	Y	Y	Y

Results

Studies Selected and Characteristics

In the initial phase of selection for our systematic review, we identified 6376 studies for abstract review. After discarding duplicates, we found 179 studies that qualified for full-text review. Four studies met the initial inclusion criteria for our systematic review [15-18]. Study characteristics from the final included papers are summarized in Table 3. We included two prospective case series, one mixed methods prospective cohort, and one retrospective cohort. Right heart catheterization was the gold standard for diagnosing PH in all our included trials. 

**Table 3:** Study Characteristics RV: right ventricular; JVP: jugular venous pressure; P2: pulmonic component of the second heart sound

Author/year	Type of study	Study setting	Number of participants	Median Age	% Male	Physical examination tests studied	Reference test (gold standard)
Pilatis et al., 2000 [15]	Retrospective cohort	Liver failure clinic	55	47	55	RV heave and loud P2	Right heart catheterization
Solverson et al., 2016 [16]	Prospective case series	Pulmonary hypertension clinic	105	61	34	RV heave, loud P2, palpable P2, JVP > 3 cm	Right heart catheterization
Colman et al., 2014 [17]	Mixed methods cohort	Heart catheterization clinic	52	65	58	RV heave and loud P2	Right heart catheterization
Braganza et al., 2019 [18]	Prospective case series	Pulmonary hypertension clinic	116	62	40	RV heave, loud P2, palpable P2, JVP > 3 cm	Right heart catheterization

Outcomes

Pooled data for sensitivity and specificity were used to calculate DOR and likelihood ratios, which are reported for RV heave, a loud P2, JVP > 3 cm above the sternal angle, and a palpable P2 (Table 1). The former three have a pooled diagnostic odds ratio greater than one, showing that those examinations are correctly discriminating the diagnosis of PH. Palpable P2 has a pooled diagnostic odds ratio less than one, but its 95% confidence interval contains one, so that test is less likely to give conclusive information based on the studies included in this systematic review.

Quality Assessment

Our quality assessment and risk of bias are outlined in Table 2 and Table 4, respectively. The quality of our included studies overall appears to be favorable. Of note, the intervention and outcome measures were consistent across the spectrum of the trials. Specifically, all patients in all the studies received a physical examination prior to a right heart catheterization with the results being blinded to the examiners. Since our included studies were case series and cohort studies, the quality of level of evidence from our included studies is a level III according to the Oxford Centre for Evidence-Based Medicine rating system [19]. The risk of bias was moderate. Selection bias was inherent as all patients in our studies were chosen to be studied after they were referred for the same intervention, which was a right heart catheterization. Performance bias was high since all examiners in all the included trials had knowledge of the allocated intervention (right heart catheterization). Detection bias was low as all the examiners were blinded to the results of the outcomes of the allocated intervention. 

**Table 4:** Risk of Bias

Bias type	Pilatis et al., 2000 [15]	Solverson et al., 2016 [16]	Colman et al., 2014 [17]	Braganza et al., 2019 [18]
Selection bias comparable cohorts or case series	Moderate	Low	Moderate	Low
Performance bias knowledge of allocated interventions	Low	High	High	High
Detection bias blinded outcomes Assessment	Mod	Low	Low	Low
Attrition bias incomplete outcomes reporting	Low	Moderate	Low	Low

Discussion

Our systematic review found a small body of evidence exists regarding the usefulness of the physical examination in the diagnostic evaluation of suspected pulmonary hypertension. The physical examination is often cited as the initial evaluation for a patient with suspected PH; however, we were unable to find many studies to evaluate the accuracy of physical examination in the diagnosis of PH. All four of the included studies were performed on a highly defined patient population with a high pretest probability of having PH. The diagnostic odds from pooled data from our four included studies support the use of the JVP > 3 cm above the sternal angle, the RV heave, and the loud P2 as being the best physical examination signs to support the diagnosis of PH in selected patients. The JVP > 3 cm above the sternal angle has the best DOR from pooled data from our systematic review (Table 1). The only finding having a reasonably good negative predictive value is the JVP > 3 cm above the sternal angle which has a negative likelihood ratio (LR) of 0.42 (95% CI 0.31, 0.57) (Table 1). 

The importance of our study and its findings are highlighted by the poor prognosis of pulmonary hypertension in the more advanced stages and the need for more accurate early detection when the disease is more amenable to treatment. The challenge of early diagnosis has been well documented by the protean nature of the symptoms occurring in the beginning stages. Early symptoms have been identified as decreased exercise tolerance, dyspnea on exertion, and fatigue with exercise [17]. Studies suggest that many patients with PH had symptoms for longer than two years before a confirmed diagnosis [20-22]. This supports that there is abundant room for improvement in the experience of a diagnostic evaluation when these early symptoms are present [22]. Unfortunately, none of our included studies were designed to evaluate physical examination findings and the NYHA class or stage of the PH of the patients. Guidelines exist for the diagnosis and treatment of PH [3,6-8]. There has been limited literature on an evidence-based approach for the physical examination in screening or in the early detection of this condition. Screening has been suggested for selected patients with the following conditions: systemic sclerosis, human immunodeficiency virus, congestive heart failure, anorexigenic drug users, and advanced liver disease [6,22,23]. Our study has not identified any single physical examination test to accurately confirm or exclude the diagnosis of PH, and further study will be needed to determine the role of bedside diagnosis with regard to PH. A combined approach using several physical examination tests together may prove to have better accuracy in diagnosing or excluding PH. 

The loud P2 may have the longest history for being a marker of PH and was first described by Potain in 1866 [24]. The two components of the second heart sound are accentuated and further separated by inspiration due to increased blood in the RV causing the pulmonary valve to close later than the aortic valve (also known as A2). Phonocardiography studies first identified A2 as having a greater intensity than that of P2 [25]. Phonocardiography studies also showed a louder intensity of P2 was seen in patients with PH [25]. Unfortunately, there are no clear definitions on how to determine a loud P2 and the reproducibility of this test is unknown as we found no data on interobserver reliability. In one of our included studies, specialists had higher positive likelihood ratios than general internists in identifying a loud P2 [17]. The use of JVP for the diagnosis of RV failure has been well studied and has very good interobserver reliability [26-28]. 

Limitation

Our study does have several limitations. Finding a small sample size is the most obvious limitation. We also had bias in all our included trials as all the patients were selected for the study were in groups at high risk for later stage PH based on them all being referred to specialty clinics for a right heart catheterization. Only the study by Colman et al. in 2017 had patients examined before a right heart catheterization for any reason [17]. Two of the studies were performed in PH clinics [16,18] and one in patients with advanced liver disease [15]. All patients in the four included trials received a right heart catheterization and each examiner had prior knowledge of this intervention (although blinded to the results). Blinding of the results reduces the risk that prior knowledge of intervention alone without knowledge of the results may affect outcome measurement. Thus, our studies likely only have a low degree of detection bias. There were no control groups in any of the trials. The generalizability of the examination for a loud P2 is difficult to quantify and there is no data available for interobserver reliability for this finding. The most glaring limitation of our systematic review was that none of our included studies were able to correlate physical examination findings with the patient's NYHA functional class or stage of PH. Given these limitations, we look forward to further studies, with well-designed cohorts and control groups, to expand our knowledge on the accuracy of physical examination in the diagnosis of PH.

## Conclusions

In conclusion, we performed our systematic review on the usefulness of the physical examination in the diagnosis of pulmonary hypertension. Our study found that the JVP > 3 cm above the sternal angle, a loud P2, and the RV heave are the most useful physical examination findings to diagnose PH in a small sample of patients. Only the JVP > 3 cm above the sternal angle had moderate accuracy for helping to confirm the diagnosis of PH in a very narrow range of patients who were at high risk of this diagnosis. We encourage larger cohort studies with control groups of patients using a combination of tests to possibly shed more light on the role of the physical examination in the diagnosis of pulmonary hypertension. Since many patients at high risk for developing pulmonary hypertension have several opportunities each year to be examined by physicians, a strategy to improve early detection may result in increasing the number of patients diagnosed at earlier stages which may help to improve outcomes.

## Appendices

Study protocol

Our study was a systematic review of the existing literature. Statistical analysis of these findings were performed to become a meta-analysis. This study is exempt from approval by our institutional review board.

Several different physical examination techniques have been described to be useful in assisting in the clinical diagnosis of pulmonary hypertension. We aimed to perform a systematic review and meta-analysis to identify the best evidence for accuracy in physical examination to assist in the clinical diagnosis of pulmonary hypertension. We included all physical maneuvers used to diagnose pulmonary hypertension.

We formed the study (PICOS) question as follows: for patients being evaluated for suspected pulmonary can the physical examination be reliably accurate in identifying patients who may need further studies to confirm the suspected diagnosis. Finding tests with the highest accuracy would require a comparison with a "gold standard" diagnostic test. The gold standard for our comparison for the diagnosis of PH is the right heart catheterization.
